# Prevalence, Determinants, and Management Options of Scar Site Pregnancy in Women With Previous Cesarean Section: A Study From the Al-Qassim Region

**DOI:** 10.7759/cureus.65874

**Published:** 2024-07-31

**Authors:** Zaheera Saadia, Khalid Nasralla, Javed Iqbal, Ebtihal Elamin

**Affiliations:** 1 Obstetrics and Gynecology, Qassim University, College of Medicine, Buraydah, SAU; 2 Obstetrics and Gynecology, Maternity and Children Hospital, Buraydah, SAU

**Keywords:** methotrexate, ultrasound, aspiration, ectopic pregnancy, scar site pregnancy

## Abstract

Introduction: Pregnancy located outside the uterine cavity following a cesarean section has become more prevalent in recent years due to the increase in cesarean section delivery. This study sought to investigate the prevalence, determinants, and treatment options of scar site pregnancy among women who sought maternal and child health services in a hospital in Buraydah, Al-Qassim region, Saudi Arabia.

Methods: Utilizing a quantitative retrospective case-control design, 50 women were recruited and assigned to the two groups evenly. Demographic data and risk factors were assessed using a questionnaire, and data were analyzed using SPSS version 27 at a 95% confidence interval and presented in tables and figures.

Results: Fifty-eight percent of the participants were aged 35 years and above, with 38% reporting a parity of 1-3. Logistic regression revealed that parity (odds ratio (OR) = 10.975, 95% confidence interval (CI) = 0.887-135.861, and p-value = 0.062), the interval between the last and present pregnancies (OR = 0.056, 95% CI = 0.005-0.668, p-value = 0.023), intrauterine contraceptive device (IUCD) use in the last year (OR = 0.070, 95% CI = 0.006 -0.780, p-value = 0.031) were statistically significant in predicting cesarean scar pregnancy. Combined methotrexate and aspiration were the most prevalent treatment options for scar site pregnancy in this study.

Conclusion: Scar site pregnancy is a maternal health complication that affects women across all healthcare settings, and its prevalence is not clear due to underdiagnosis and underreporting. The risk of scar site pregnancy increased with an increase in the number of childbirths (parity) and the interval between the last and current pregnancies.

## Introduction

Implantation of conceptus outside the uterine cavity is mostly seen in fallopian tubes, ovaries, cervix, or abdominal cavity [1-3]. One of the rare types of ectopic pregnancy is the implantation of a gestation sac in the previous scar of cesarean section [4]. It is defined as “an abnormal implantation of the gestational sac in the area of the prior cesarean delivery scar, potentially leading to life-threatening complications, including severe hemorrhage, uterine rupture, and development of placenta accreta spectrum disorders” [5].

Previous cesarean section is a risk factor for scar site pregnancy [6]. The presentation of cesarean scar pregnancy varies among individuals and, if left untreated, can lead to catastrophic events like severe bleeding, placenta accrete, uterine rupture, and fatalities [7-8].

There is no single standardized treatment for scar site pregnancy, with reports of the use of methotrexate, intrauterine potassium chloride, aspiration, and hysterectomy [4,9-11].

The incidence of cesarean scar pregnancy has increased due to the increasing number of cesarean births [1]. Grechukhina et al. [12] estimated that the incidence of cesarean scar pregnancy ranges between one in 1,800 and one in 2,200 pregnancies. While overall ruptured ectopic from other sites has led to a maternal death rate of 2.7% [8], the exact mortality from scar-related pregnancy is unknown.

This increase in cesarean section rate has resulted in a higher prevalence of complications for repeat cesarean sections, including scar site pregnancies, which have become a gynecology and obstetrics concern today [5]. Scar site pregnancies can result in complications, such as uterine rupture, abnormal placental implantation, adherent placenta, uncontrolled hemorrhage, infertility, and sometimes death [7-8]. The prevalence of cesarean section in Saudi Arabia has increased more than threefold the World Health Organization recommendations(15%). Saudi Arabia has been reported to have a cesarean section rate ranging from 10.6% in 1997 to 19.1%, which is an increase of around 80% [8]. A study from the Al-Qassim region has reported an incidence of 55.4% for cesarean deliveries between August and October 2016, and it was reported that 42% (396 of 936) of all deliveries were cesarean sections, and repeat cesarean contributed to 21.1% of these cesarean sections [13]. This increase in cesarean rate has led to hospitals seeing more cases of scar site pregnancies, which is a primer to maternal complications, such as hemorrhage. However, there is a paucity of literature on the prevalence, determinants, and management options of scar site pregnancy in women with previous cesarean section in this study’s geographical area. Therefore, this study aims to assess the prevalence, determinants, and management options for scar site pregnancy in women with previous cesarean deliveries in the Al-Qassim region of Saudi Arabia. Findings will provide data-driven insights on this condition, which will inform clinical practice and management options, reducing maternal morbidity and mortality.

The increase in global and regional cesarean sections and, subsequently, scar site pregnancies and placenta previa has greatly increased maternal morbidity and mortality. Identifying the risk factors and timely appropriate management is key to averting maternal complications among women with a history of cesarean section. The findings of this study are useful to the field of obstetric practice and local policy formulation on the management options of scar site pregnancy in women with previous cesarean section in the Al-Qassim region, Saudi Arabia.

## Materials and methods

Study design, setting, and duration

This is a retrospective case-control study conducted at Maternity and Children Hospital, Buraydah, Al-Qassim, Saudi Arabia.

Using patient records obtained from the department (January 2023 to December 2023), participants were categorized into two groups, i.e., cases and control.

Cases were assigned based on ultrasound and color Doppler for scar site pregnancy. These patients presented in the first trimester, while the control group was women who had their pregnancies terminated at term for a repeat cesarean section, due to placenta previa.

All women who had a history of cesarean section but did not carry the subsequent pregnancy to term and those whose pregnancy at term was terminated due to reasons other than placenta previa were excluded from the study. Women with multiple cesarean sections were also excluded from the study population.

Using the simple random sampling technique, 50 women were recruited to take part in the study.

Study variables and data collection

Data were collected using structured questionnaires on demographic characteristics, determinants of scar site pregnancy, and management options (see Appendix). The data collected were anonymized by removing all identifiers and stored securely in a password-encrypted drive. Data analysis was done using IBM SPSS Statistics, version 27.0 (released 2020, IBM Corp., Armonk, NY) at a 95% confidence interval, and the results were presented in narrative, tables, and figures.

Variables studied were age, gender, gravidity, parity, previous abortion, the interval between the last pregnancy and current pregnancy, use of the intrauterine contraceptive device (IUCD), history of pelvic inflammatory disease (PID), and pregnancy by assisted reproductive techniques (ART). Clinical presentations like pain abdomen, syncope, and bleeding and treatment options used like intramuscular methotrexate, methotrexate, or potassium chloride (KCL) in the intrauterine gestational sac, aspiration, and dilatation and curettage.

Statistical analysis

A chi-square test of association was used to compare the determinants of scar site pregnancy between the cases and controls. Binary logistic regression was applied to identify the determinants of scar site pregnancy and to examine which determinants helped explain whether a pregnancy will end up in scar site pregnancy.

Ethical approval

Ethical approval to conduct this research was sought from the Institutional Review Board of Qassim University (ref. no. CL-20240-30) and the Ministry of Health, General Directorate of Health Affairs, Al-Qassim region (ref. no. 607/45/10564). In addition to consent, confidentiality, respect for autonomy, justice, beneficence, and non-maleficence were guaranteed for all the participants. 

## Results

Demographic characteristics

This study recruited 50 participants with a history of previous one cesarean section; 25 women were assigned to the case and control groups each. For the case group, 15 (60%) were aged 35 and above. Sixteen (64%) women reported gravidity above 5, and nine (36%) reported a parity of 5 or more. Forteen (56%) had one to two abortions, and the interval between the current and last pregnancies was two to five years for 14 (56%) women. Seventeen (68%) reported not having used IUCD after the last childbirth, 17 (68%) reported no history of PID, 22 (88%) reported no history of urinary tract infection (UTI) since the last birth, and 20 (80%) did not report a current pregnancy by ART.

For the control group, 14 (56%) were aged 35 and above. Fifteen (60%) women reported gravidity above 5, and 11 (44%) reported a parity of 3 and below. Thirteen (52%) had no history of abortions, while 12 (48%) reported one to two abortions. The interval between the current and last pregnancies was two to five years for 16 (64%) women, 23 (92%) reported not having used IUCD after the last childbirth, and 21 (84%) reported no history of PID. All women in the control group reported no history of UTI since their last birth and that the current pregnancy was not by ART, as summarized in Table 1. Majority of women diagnosed with scar site pregnancy presented with abdominal pain (n = 16), vaginal bleeding (n = 15), and both abdominal pain and vaginal bleeding (n = 15), as shown in Table 1.

**Table 1:** Demographic characteristics PID: pelvic inflammatory disease, UTI: urinary tract infection

Demographic characteristics		Case group n = 25 (%)	Control group n = 25 (%)
Maternal age	Below 35 years	10 (40)	11 (44)
	35 years and above	15 (60)	14 (56)
Gravidity	1-3	1 (4)	3 (12)
	4-5	8 (32)	7 (28)
	Above 5	16 (64)	15 (60)
Parity	1-3	8 (32)	11 (44)
	4-5	8 (32)	8 (32)
	Above 5	9 (36)	6 (24)
Previous miscarriages	None	8 (32)	13 (52)
	1-2	14 (56)	12 (48)
	3 and above	3 (12)	0 (0)
Current pregnancy by ART	Yes	5 (20)	0 (0)
History of PID	Yes	8 (32)	4 (16)
History of UTI since last birth	Yes	3 (12)	0 (0)
Clinical presentation	Asymptomatic	1 (4)	2(8)
	Referred from BHU	1 (4)	0 (0)
	Pain abdomen	9 (36)	7 (28)
	Vaginal bleeding	5 (20)	10 (40)
	Both abdominal pain and vaginal bleeding	9 (36)	6 (24)

A chi-square test of association was run to determine the association between the independent and dependent variables. The relationship between cesarean section pregnancies and used IUCD after last birth was significant (χ2 = 4.500, p = 0.034), indicating that pregnancies of women who used IUCD after last birth were more likely to end up in scar site pregnancy than term pregnancy. Likewise, pregnancies of women who used ART for conception were more likely to end up in scar site pregnancy than term pregnancy (χ2 = 5.556, p = 0.050), as shown in Table 2.

**Table 2:** Determinants of scar site pregnancy F: Fisher test value taken, PID: pelvic inflammatory disease, UTI: urinary tract infection, ART: artificial reproductive technique, LSCS: lower segment cesarean section

Determinants of scar site pregnancy		Case group n = 25 (%)	Control group n = 25 (%)	Χ²	P-value
Used IUCD after the last birth	Yes	8 (32)	2 (8)	4.500	0.034
	No	17 (68)	23 (92)		
History of PID	Yes	8 (32)	4 (16)	1.781	0.182
	No	17 (68)	21(84)		
History of UTI since the last birth	Yes	3 (12)	0 (0)	1.63	0.235
	No	22 (88)	25 (100)		
Current pregnancy by ART	Yes	5 (20)	0 (0)	5.556	(0.050)F*
	No	20 (80)	25 (100)		
Previous LSCS interval in years	Below 5	24 (96)	23 (92)	0.361	0.548 F*
	Above 5	1 (4)	2 (8)		
	Below 1	9 (36)	7 (28)	0.368	0.544
	Above 5	16 (64)	18 (72)		
	Interval 2-5	14 (56)	23 (92)	8.420	0.004
	Above 5	11 (44)	2 (8)		
Gravidity	Below 5	9 (36)	10 (40)	0.085	0.771
	5 and above	16 (64)	15 (600		
Age	Below 35	10 (40)	11 (44)	0.082	0.774
	35 and above	15 (60)	14 (56)		
Previous miscarriages	none	8 (32)	13 (52)	2.053	0.152
	1-3	17 (68)	12		

A binary logistic regression was conducted to examine whether maternal age, gravidity, parity, abortions, the interval between the current pregnancy and last pregnancy, using IUCD after the last birth, H/O PID, and H/O UTI since the last birth and current pregnancy by ART helped explain whether a pregnancy will end up in scar site pregnancy. The data met the binary logistic regression assumptions of independent observations, no perfect multicollinearity, and a linear relationship. The model was statistically significant at explaining the determinants of scar site pregnancy than the baseline (X2 (10) = 34.687, p < 0.001). The model correctly classified 80% of the cases, including 76% of women who had scar site pregnancies and 84% who had term pregnancies.

The interval between the current and last pregnancies and IUCD use were significant predictors for scar site pregnancy as compared to term pregnancy (Table 3).

**Table 3:** Model summary for binary logistic regression of scar site pregnancy and demographic variables PID: pelvic inflammatory disease, UTI: urinary tract infection, ART: artificial reproductive technique, LSCS: lower segment cesarean section

Step 1a	Demographic variables	B	S.E	Wald	df	Sig.	Exp(B)	95% CI for lower	95% Ci for upper
	Maternal age	-1.488	1.519	0.960	1	0.327	0.226	0.12	4.430
	Gravidity	-0.795	1.258	0.399	1	0.528	0.452	0.038	5.320
	Parity	2.396	1.284	3.483	1	0.062	10.975	0.887	135.861
	Abortions	1.239	0.997	1.543	1	0.214	3.451	0.489	24.361
	Interval between the current and last pregnancies	-2.878	1.262	5.197	1	0.023	0.056	0.005	0.668
	Indication of the last LSCS	-0.292	0.299	0.954	1	0.329	0.747	0.415	1.342
	Used IUCD after the last birth	-2.663	1.232	4.673	1	0.031	0.070	0.006	0.780
	History of PID	-1.251	1.383	0.819	1	0.365	0.286	0.019	4.300
	History of UTI since the last birth	-24.301	21976.128	0.000	1	0.999	0.000	0.000	-
	Current pregnancy by ART	-23.122	16258.977	0.000	1	0.999	0.000	0.000	-
	Constant	104.955	54637.777	0.000	1	0.999	3.813E=45	-	-

The interval between the last and current pregnancies (with 95% CI = 0.005-0.668 and p-value = 0.023) was a predictor of scar site pregnancy. Exp B was -2.878, revealing that for each unit increase in the interval between pregnancies, the chance of scar site pregnancy was less by an Exp of -2.878. Thus, a shorter interval between the current and last pregnancies was associated with higher odds of scar site pregnancy.

IUCD use after the last pregnancy also had a negative association ( 95% CI = 0.006-0.78, p-value = 0.031). Exp B was -2.663, revealing that using IUCD after the last pregnancy lowers the chances of scar site pregnancy.

However, the odds of maternal age (OR = 0.226, 95% CI = 0.012-4.430), gravidity (OR = 0.452, 95% CI = 0.038-5.320), H/O PID (OR = 0.286, 95% CI = 0.019-4.300), H/O UTI since last birth (OR = 0.000, 95% CI = 0.000), and current pregnancy by ART (OR = 0.000, 95% CI = 0.000) predicting scar site pregnancy were less than those predicting term pregnancy (Table 3).

Treatment options for scar site pregnancy

This study explored the treatment options available for scar site pregnancy; the options investigated were methotrexate, transvaginal aspiration, dilation and curettage, laparoscopic resection, and KCL intrauterine. The most common treatment option for scar site pregnancy was combined I‎/M (intramuscular) methotrexate and aspiration (n = 12, 48%), while the least was KCL intrauterine (n = 1, 4%). Notably, laparoscopic resection, dilation, and curettage were never used as a treatment option for this sample population (Table 4).

**Table 4:** Treatment options for scar site pregnancy KCL: potassium chloride

Treatment options
Intra muscular methotrexate
Transvaginal aspiration
Combined intramuscular methotrexate and aspiration
No treatment
KCL intrauterine
Dilation and curettage
Laparoscopic resection

## Discussion

Given how infrequently cesarean scar pregnancy cases have been documented in the literature, the prevalence is unknown. In their local community of women visiting the early pregnancy assessment unit, Jurkovic et al. [6] estimated a prevalence of 1:1800. A recent case series estimated an incidence of 1:2226 of all pregnancies, with a rate of 6.1% of all ectopic pregnancies in women who had at least one cesarean delivery and 0.15% in women who had a previous cesarean section. Ammar et al. [14] noted that in the UAE, the rate of cesarean sections in tertiary hospitals is approximately 30%, and at least 6% of ectopic pregnancies diagnosed as cesarean scar pregnancy are among women, with a history of a cesarean scar. Since the first diagnosis in 1978 [15], cesarean scar pregnancy has increased over the years as a result of the increase in repeat cesarean sections, which carry the risk of maternal complications. Women with a history of cesarean section are likely to experience ectopic pregnancies because of scarring and damage to the uterine lining [6].

The cases of scar site pregnancy among women receiving obstetrics and gynecology services at Maternity and Children Hospital, Buraydah, which is located in the Al-Qassim region, Saudi Arabia, was 25 during the time period of January 2023 to December 2023. However, the prevalence of scar site pregnancy in this population could not be determined because we could not obtain the definite size of the population at risk. The CS prevalence rates in two tertiary healthcare facilities in Dubai and Qassim regions were 30% (2015-2017) and 42% (August to October 2016), respectively [13-14], denoting that the overall prevalence was higher than the WHO ideal recommendation of 15% [16]. This rising rate of cesarean section could be attributed to the higher prevalence of scar site pregnancies. Grechukhina et al. [12] estimated that the incidence of cesarean scar pregnancy ranged between one in 1,800 and one in 2,200 pregnancies among women diagnosed from May 2013 to March 2018 at Yale-New Haven Hospital.

The risk of scar site pregnancy increases with the increase in maternal age because elder mothers have fixed pelvises, making vaginal delivery more difficult and subjecting them to CS procedures, which prevent endometrial and associated scars from healing well. There are mixed findings of association between maternal age and scar site pregnancy. Chow et al. [3] found that the risk of ectopic pregnancy increases as the maternal age increases. Similarly, Zhou et al. [16] concluded that maternal age was an independent risk factor for cesarean scar pregnancy. This is in contrast to the results of our study where we found age as not a good predictor of scar site pregnancy over term pregnancy (Table 3).

Parity and number of previous abortions have also been reported to increase the risks of scar site pregnancy. Parity may contribute to scar site pregnancy because of abnormalities caused by repeated uterus scarring and healing. Multiple abortions damage the uterus endometrium, compromise the integrity of the anterior uterine wall, and increase the risk of scar site pregnancy. Gravidity, parity, and number of abortions increase the risk of scar site pregnancy [16-18]. However, Chow et al. found no evidence that the number of abortions and gravidity increases the risk of an ectopic pregnancy [3]. The present study found that the chances of scar site pregnancies increase with every additional childbirth because each subsequent cesarean delivery weakens the uterine wall, allowing easier implantation of the embryo in the scar site (Table 3). The findings of this study are consistent with those of Zhang et al. [17] and Chow et al. [3], which noted higher rates of scar site pregnancies in women with higher parity.

PID is a significant risk factor for scar site pregnancy because, if untreated, it may allow scar tissue to develop in the fallopian tubes, preventing the fertilized egg from passing the fallopian tube on its way to the uterus for implantation. Weström et al. [19] and Gonzalez and Waxman found strong evidence of an association between PID and ectopic pregnancy. Our study did not find PID to be a significant predictor of scar site pregnancy (Table 3).

As regards ART, while applying chi-square χ2 = 5.556 and p = 0.050, it was significant to have scar site pregnancy after ART (Table 2), but applying logistic regression in the current pregnancy by ART did not predict scar site pregnancy in our study, most probably due to the small sample size. There are reports that IVF also increases the risk of scar site pregnancy [20]. Pregnancies resulting from ART have an increased likelihood of scar implantation since the endometrium is disturbed in the process of transferring the embryo [3]. However, Zhang et al. failed to find an association between scar site pregnancy and current pregnancy through ART [21].

IUCD use in the last year (OR = 0.070, 95% CI = 0.006-0.780, p-value = 0.031) was statistically significant in predicting cesarean scar pregnancy. As the number of years increases, the possibility of IUCD becomes a poor predictor of scar site pregnancy. Using IUCD after the last pregnancy increases the interval between the last pregnancies and may be a factor in lowering the risk of scar site pregnancy. Increased scar site pregnancy through uterus injury may occur during insertion or removal and inflammation caused by the device. Majangara et al.'s findings support that using IUCD increases the risk of scar site pregnancy [22]. Using IUCD may cause inflammation, blocking the embryo from making its way to the uterus. These findings are not consistent with those of others [23].

This study observed that the interval between the last and current pregnancies(95% CI = 0.005-0.668, p-value = 0.023) was a predictor of scar site pregnancy. Exp B was -2.878, revealing that for each unit increase in the interval between pregnancies, the chance of scar site pregnancy was less by an Exp of -2.878. A short interval between the current and last pregnancies would fuel the risk of a scar site pregnancy, especially if the last resulted in a cesarean section because the uterine scar may not have healed, and the scar tissue is still thin and weak. Zhou et al. [16] found the interval between the current pregnancy and the last CS to be a significant risk factor for cesarean scar pregnancy. However, according to Ash et al. [24], there is no relationship between the Interval between current pregnancy and last pregnancy and scar site pregnancy.

Finally, a history of UTI can coexist with PID and enable inflammation and scar tissue to form along the uterine wall, allowing the embryo to be implanted in the scar site [19]. Other known risk factors of scar site pregnancy, including abortions, indication of last LSCS, H/O PID, and H/O UTI, may cause damage and weaken the endometrium, making it easier for the embryo to be implanted at the scar site. However, we could not find them to be a better predictor of scar site pregnancy.

Bleeding was a common presentation among women presenting with scar site pregnancy [7,8]. In this study, the majority of women presented with vaginal bleeding (30%), abdominal pain (32%), and both vaginal bleeding and abdominal pain (30%), which is in contrast to De Braud et al. [25], who investigated 62 viable cesarean scar pregnancies from a single center and found that 26% of women were asymptomatic, 23% presented with vaginal bleeding, 12% with abdominal pain, and 39% with a combination of both. Similarly, a retrospective cohort of 232 cesarean scar pregnancies by Jurkovic et al. [26] found that 24.5% of women were asymptomatic, 48.5% presented with vaginal bleeding, 9% with abdominal pain, and 18% with a combination of both. Given this data, it is possible that a quarter of women who remain asymptomatic with cesarean scar pregnancy may go undiagnosed in the first trimester. Evidently, vaginal bleeding and abdominal pain or both are the most prevalent clinical manifestations of scar site pregnancy.

The management options for scar site pregnancy vary from medical to surgical, depending on its size and type. It is therefore recommended that cesarean scar pregnancy treatment should be initiated soon after diagnosis, preferably in the first trimester, to avert any maternal complication [27]. However, there is no single standardized treatment for scar site pregnancy [6], with reports of the use of methotrexate, intrauterine potassium chloride, aspiration, and hysterectomy advanced for the management of patients by different obstetrics and gynecology specialists.

Methotrexate is the standard treatment for tubal ectopic pregnancy because of its efficacy when used below eight weeks of pregnancy with HCG levels of less than 5000 IU [14]. Methotrexate can also be offered as a combined local and systemic methotrexate. Patients undergoing this treatment should meet certain medical criteria before the treatment is initiated. The prerequisites for methotrexate treatment are full blood count, no severe or persistent abdominal pain, normal baseline liver and renal function test results, and commitment to follow-up until the ectopic pregnancy has resolved [24-28].

Transvaginal aspiration involves the removal of tissue or fluid from the body through the vagina. Transvaginal aspiration minimizes the potential damage to the uterus, thereby preserving the woman's reproductive capacity. This consideration is particularly important for women who desire future pregnancies and wish to maintain their fertility despite facing the challenges posed by scar-site pregnancies. Transvaginal aspiration can be performed as an outpatient procedure in many cases, reducing the burden on healthcare resources and minimizing the need for hospitalization.

Combined methotrexate and transvaginal aspiration management of scar site pregnancies presents a comprehensive approach to address the unique challenges posed by ectopic pregnancies. This treatment methodology involves the administration of methotrexate, an antimetabolite, followed by transvaginal aspiration to remove trophoblastic tissue or fluid. The combination aims to provide an effective and minimally invasive solution while preserving fertility and ensuring patient safety.

KCL intrauterine involves the injection of potassium chloride directly into the gestational sac. Studies evaluating the use of KCL intrauterine instillation for CSP management have reported varying success rates. While some studies suggest favorable outcomes with reduced complications, others emphasize the need for further research to establish its efficacy and safety conclusively. Concerns include the risk of infection, incomplete pregnancy resolution, and potential complications associated with the injection procedure.

Surgical interventions for the management of scar site pregnancy include laparoscopic resection, dilation and curettage, and hysterectomy. Dilation and curettage are not preferred first-line treatment options because they bear a high risk of failure and post-operative complications. Prior to the procedure, the distance from the bladder and the thickness of the scar must be determined. Tekin et al. [29] recommended dilatation and curettage as an option of treatment in early cases of CSP in which the myometrium thickness was >4.5 mm.

Combined therapy is preferred for its significant reduction in the risk of adverse events. According to Giampaolino et al. [30], the use of methotrexate alone, dilation and curettage, and the administration of intramuscular methotrexate combined with dilation and curettage bore the highest risk of adverse events, when used to treat cesarean scar pregnancy. Therefore, combined therapy using I/M methotrexate and aspiration is the most preferred treatment option in comparison to the single-treatment option [30]. In a similar study, methotrexate used alone had a success rate of 74%, while methotrexate used in combination with another treatment option achieved a success rate of 86% [14], making combined therapy the most used in retrospect.

The main limitation of this study was the small sample and questionnaire-based data collection that was used, thus making it difficult to generalize the findings.

## Conclusions

Scar site pregnancy is a maternal health complication that affects women across all healthcare settings and whose prevalence is not clear due to underdiagnosis and underreporting. The risk of scar site pregnancy increased with an increase in the number of childbirths (parity) and the interval between the last and current pregnancies. Combined therapy (combined I/M methotrexate and aspiration) was the most prevalent treatment option for women who received obstetrics and gynecology services at Maternity and Children Hospital, Buraydah. Following the small sample size, this study recommends that a similar study be conducted using a bigger sample size to test the significance levels of the risk factors for scar site pregnancy among women of childbearing age.

## Appendices

Study questionnaire

Maternal Data

Patient ID--------------------    

Group 1-cases----(Scar site pregnancy)

Group 2- control (Placenta Previa with previous C-section)

Demographic factors

Maternal Age-----------------------

Gravidity-------------

 Parity --------------

Previous miscarriages-------------

Interval between current pregnancy and last LSCS----

Indication of last LSCS--------

Used IUCD after last LSCS----------

H/OID------------

H/O UTI---------

Current pregnancy by ART----------

Clinical presentation

Asymptomatic

Referred from BHU after diagnosis of Placenta previa or scar site pregnancy

Pain abdomen

Vaginal bleeding

Both abdominal pain and vaginal bleeding

Treatment

I/M methotrexate

Transvaginal aspiration

Combine methotrexate and aspiration

No treatment

KCL intrauterine

Dilatation and curettage

Laparoscopic resection
